# Assessing service availability and readiness of healthcare facilities to manage diabetes mellitus in Bangladesh: Findings from a nationwide survey

**DOI:** 10.1371/journal.pone.0263259

**Published:** 2022-02-16

**Authors:** Hasina Akhter Chowdhury, Progga Paromita, Cinderella Akbar Mayaboti, Shagoofa Rakhshanda, Farah Naz Rahman, Minhazul Abedin, A. K. M. Fazlur Rahman, Saidur Rahman Mashreky

**Affiliations:** 1 Centre for Injury Prevention and Research, Bangladesh (CIPRB), Dhaka, Bangladesh; 2 Department of Epidemiology and Preventive Medicine, School of Public Health and Preventive Medicine, Monash University, Melbourne, Australia; 3 Kirtipasha Health and Family Welfare Centre, Jhalokathi Sadar Upazila, Barishal, Bangladesh; University of Dhaka: Dhaka University, BANGLADESH

## Abstract

**Introduction:**

Diabetes Mellitus (DM) is one of the most prevalent non-communicable diseases (NCDs)as well as a major cause of morbidity and mortality worldwide. Around 80% diabetic patients live in low- and middle-income countries. In Bangladesh, there is a scarcity of data on the quality of DM management within health facilities. This study aims to describe service availability and readiness for DM at all tiers of health facilities using the World Health Organization’s (WHO) Service Availability and Readiness Assessment (SARA) standard tool.

**Methods:**

This cross-sectional survey was conducted in 266 health facilities all across Bangladesh using the WHO SARA standard tool. Descriptive analyses for the availability of DM services was carried out. Composite scores for facility readiness index (RI) were calculated in four domains: staff and guideline, basic equipment, diagnostic capacity, and essential medicines. Indices were stratified by facility level and a cut off value of 70% was considered as ‘ready’ to manage diabetes at each facility level.

**Results:**

The mean RI score of tertiary and specialized hospitals was above the cutoff value of 70% (RI: 79%), whereas for District Hospitals (DHs), Upazila Health Complexes (UHCs) and NGO and Private hospitals the RI scores were other levels of 65%, 51% and 62% respectively. This indicating that only the tertiary level of health facilities was ready to manage DM. However, it has been observed that the RI scores of the essential medicine domain was low at all levels of health facilities including tertiary-level.

**Conclusions:**

The study revealed only tertiary level facilities were ready to manage DM. However, like other facilities, they require an adequate supply of essential medicines. Alongside the inadequate supply of medicines, shortage of trained staff and unavailability of guidelines on the diagnosis and treatment of DM also contributed to the low RI score for rest of the facilities.

## Introduction

Diabetes Mellitus, one of the most prevalent Non communicable diseases (NCDs) globally, poses an increasing risk of premature death and disability [1,2]. It causes crucial metabolic changes that increase the risk of other NCDs [3]. Accordingly, diabetes mellitus (DM) is a serious and costly public health issue targeted for action by world leaders as the global prevalence of this disease is increasing at an alarming rate. The International Diabetes Federation (IDF) estimated about 463 million adults worldwide have diabetes, of which 79.4% live in low- and middle-income countries; and South Asians have a 3-fold higher prevalence of diabetes compared to Europeans [4,5]. In Bangladesh, currently 8.8 million people have diabetes, and projections showed it will be 15.0 million by2045, if the incidence of the disease continues to grow at the present rate [6].

Bangladesh has a pluralistic health service delivery system comprising health institutions and providers in the public (linked to each other by referral system), private for-profit, not for-profit and informal sectors [7,8]. Ensuring standard, efficient, and effective quality services to the patients is the priority of the country’s health system [9]. Bangladesh’s Ministry of Health and Family Welfare (MOHFW) has allocated considerable funds and developed several strategic plans based on surveys, policies, action plans, guidelines and strategic planning documents, with the aim to strengthen the capacity and competency of the health system for the integrated management of non-communicable diseases and their risk factors [10,11]. Early detection and management of diabetes along with other common NCDs are now an integral part of the Essential Service Package (ESP) under the Government’s fourth Health, Nutrition, Population Strategic Investment Plans (HNPSIP2016-2021), which prioritizes equal, effective and sustainable service for hard to reach and vulnerable population [12,13]. Currently in Bangladesh, health education and screening for diabetes are being provided even at community level, whereas treatment facilities are limited to the Upazila level [13].

Despite substantial progress in the management for the prevention or postpontment of diabetes and its complications, the outcome of diabetic care in Bangladesh is still far from optimal [14]. For improvingthis situation, it is now imperative to generate evidence but research on related issuesthe context of is still scarce, andstudies designed to explore issues of health system that contribute to care of diabetes from a public health perspective is also scant. Moreover, there are inadequate evidences regarding service availability and preparedness for DM.

To address the growing burden of NCDs, including diabetes, more systematic and methodically robust approaches are required [15,16]. Few studies have been conducted to assess the situation of healthcare facilities in Bangladesh in terms of their capacity to manage diabetes [16]. These studies, however, had limitation for generalizability due to their relatively smaller sample size and lack of inclusion of tertiary healthcare facilities. A comprehensive assessment of healthcare facilities’ readiness to manage diabetes on a national scale is required to identify existing challenges and gaps in services and to develop a strategy for strengthening the health system. Against this backdrop, the current study aimed to generate evidence on the availability of services and the readiness of healthcare facilities to manage diabetes in Bangladesh, using the WHO SARA tool. The current study evaluated tertiary healthcare facilities in this regard for the first time, and identified specific domains in the health system that require improvement in order to establish adequate service provision for diabetes management across the country.

## Methods

### Design, sampling, and sample size

The study deployed a cross-sectional facility-based survey. Randomly selected healthcare facilities (both public and private) from all tiers of the health system across the seven divisions of the country were included in this study conducted. The time period was between December 2017 to June 2018.

The current study is the part of a larger study called ‘Service Availability and Readiness Assessment (SARA) Survey for NCDs and Disability Service Delivery System in Bangladesh’ using WHO SARA Tool [17], which surveyed 590 health facilities in Bangladesh. From this, relevant facilities for diabetic care have been included for analysis in this study.

**Fig 1:**
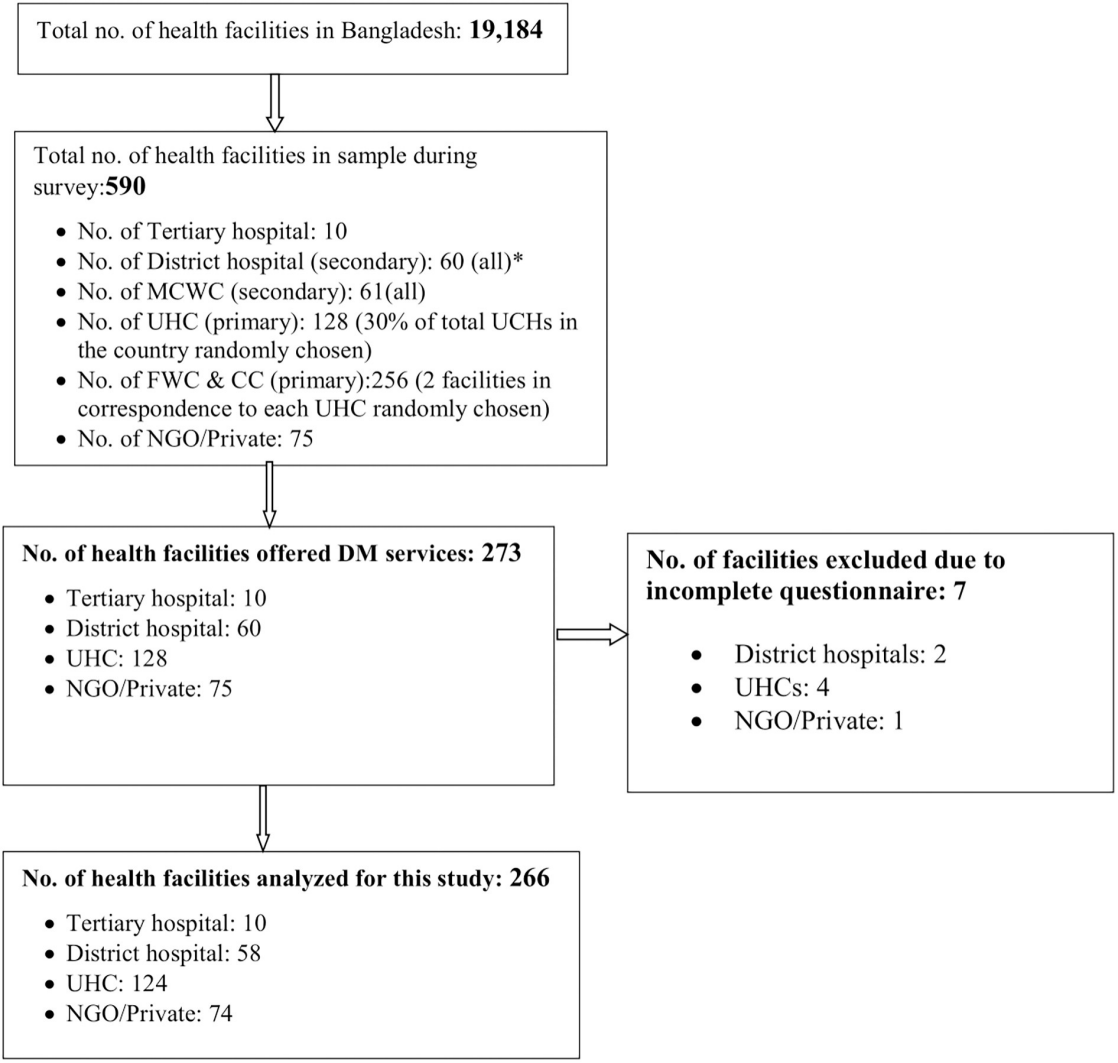
Sampling and study inclusion flow chart. [UHC: Upazila Health Complex, NGO: Non-Government Organization.*rest of the district hospitals had been converted to medical college hospital (tertiary facilities)].

From the Bangladesh Health Facility Survey (BHFS) 2014, National Institute of Population Research and Training (NIPORT)a list of all health facilities in seven divisions of Bangladesh was obtained. These included public, private and NGO health facilities. A total of 19184 health facilities were identified, which were used as the sampling frame for the parent study [Fig 1]. In this study, at primary level: Community Clinics (CCs), Family Welfare Centers (FWCs), and Upazila Health Complexes (UHCs), at secondary level: District Hospitals (DHs) and Mother and Child Welfare Centres (MCWCs), and at tertiary level: medical colleges and specialized hospitals were considered. Using the NIPORT sampling frame, the sample size for different level of health facilities were decided based on the homogeneity of services and assumption on data saturation. The service provision and available facilities at community clinics, FWCs, and UHCs are mostly homogenous across the country. Therefore, it was assumed that randomly selecting two community clinics from each Upazila, and two UHCs from each district, would be adequate to provide a generalized picture. For the UHCs and FWCs, the required number was also decided considering few geographical variations. Since service delivery and facilities differ substantially in the secondary and tertiary level health facilities across different regions of the country, we included all 60 district hospitals, 61 MCWCs, and relevant tertiary and specialized hospitals for adequate representation (that authors assumed would achieve data saturation). However, while finalizing sample size at various level of health facilities, available resources for the study were also considered, which may limit the methodological robustness of the sampling technique. It has been reported in the discussion section.

In accordance with the provision for diabetic care, the current study analyzed data from 266 facilities (out of 590 facilities in the parent study), excluding CCs, FWCs, and MCWCs, and seven other facilities which were excluded due to incomplete data.

### Data collection procedure

The WHO SARA Tool, which has been validated in Bangladesh, was used to collect data for this study. Each healthcare facility was evaluated by the SARA tool based on four domains: staff and guidelines, basic technology and equipment, diagnostic capacity, and essential medicines. The information on these four domains for a health facility was primarily gathered from the head of a facility or a management staff member with sufficient knowledge of hospital capacity and operations (Table 1). In the event that the facility’s head could not be reached, we approached the next person in the hierarchy. When reported to be available, the data collectors checked for the presence of guidelines, medicines, and diagnostic facilities to further supplement and to assess the quality of the information provided by the health authority. This observation method was also used by data collectors to assess the availability of equipment and diagnostic services.

**Table 1:** Tracer items in respective domains for diabetes mellitus services.

Domain	Tracer Items	Definition
1. Staff and Guidelines	Endocrinologist	Medical personnel
	Medicine doctor	
	At least one staff member (Nurse, Technician) providing DM services trained in some aspect of DM care	Training on diabetes management
	Guidelines for diagnosis & treatment of diabetes mellitus (DM)	Observed presence of national (& other) guidelines for DM
2. Basic technologies/ Equipment	Adult weighing scale	Observed availability & reported functionality of each item at the facility
	Measuring tape/Height Board (used for measuring height)	
	Glucometer	
	Blood pressure measurement device	
3. Diagnostic facility	Blood glucose	Able to conduct the test at the facility and observed availability of functioning equipment & reagents for the test
	Urine dipstick- protein	
	Urine dipstick- albumin/ ketones	
4. Essential medicines	Glucose 50% injection	Observed availability of each medicine at the facility
	Sulphonyl urea	
	Metformin 500mg/800mg	
	Injectable Insulin (Insulin regular, Insulin intermediate, Long acting Insulin and Insulin Analogues)	

Forty-eight interviewers were recruited, each with a Bachelor of Medicine and Bachelor of Surgery (MBBS). The interviewers, were trained for2 days on the data collection instrument and data entry into RedCap software using tablets. The interviewers received instructions for uploading the data to the server immediately after data collection from each health facility. This enabled researchers at the head office to monitor the data collection in real-time. The health facilities included in this study were contacted beforehand and an appointment was set for the interviews. The interviews were conducted in-person and were recorded for further analysis. The details of the data collection method have been fully described in studies by Paromita and Rakhshanda [18,19].

### Instrument, variables and statistical analysis

WHO SARA Standard Tool, which is a comprehensive facility-based assessment tool, was used to collect data for conducting the assessment [17]. Drawing on the WHO SARA core instrument and guidelines, the questionnaire was comprised of 15 tracer items for diabetes care distributed in four domains: a) staff & guidelines, b) basic technologies/equipment, c) diagnostics and d) essential medicines (Table 1). These four domains were compiled to calculate the readiness score.

The filled-up questionnaires in the software were checked meticulously and verified to reduce errors. The study outcome variables, ‘availability’ and ‘readiness’, were assessed for 266 facilities rendering services for diabetic care distributed in seven divisions of the country. The outcome ‘readiness’ was a composite measure of the capacity of the facilities to provide management of diabetes [20]. Readiness indicator is comprised of above mentioned 4 domains and each domain consists of a set of tracer items. The service readiness was assessed in four stages: a) Determining the availability of diabetes care service readiness indicators at each facility level; b) Calculating the tracer item index scores (number of tracer item present *100/number of tracer items that should be present); c) calculating the readiness index (RI) of facilities according to all 4 domains (the mean of all tracer item index scores in each domain); and d) calculating the facility level’s mean readiness score (the average of the readiness index of all 4 domains). Indices were stratified by facility level and compared to a RI cutoff score of 70%. This cutoff was based upon a study conducted by Wilbroad Mutale *et al* in Zambia utilizing the SARA tool, which considered a facility to be ‘ready’ to manage diabetes care if it scored above 70% [18,19,21]. All data were analyzed using SPSS version 21 (SPSS, Chicago, IL).

### Ethical considerations

The ethical clearance was obtained from the Ethical Review Committee of Center for Injury Prevention and Research, Bangladesh (Reference number: *CIPRB/ERC/2017/27*). Informed written consent was taken from each respondent. Anonymity and confidentiality of the respondents were maintained throughout the data collection and analysis process.

## Results

### Overview of the surveyed health facilities for diabetes care

The results comprised of availability and readiness for management of DM among 266 healthcare facilities where 192(72.1%) were in the public health facilities. Of the public sector facilities, at the primary level were 124 (46.6%) UHCs, in the secondary level 58 (21.8%) district hospitals, and in the tertiary were10 (3.7%) facilities. More than one fourth of the facilities (27.4%) were in Dhaka division (Table 2).

**Table 2:** Mean characteristics of health facilities providing diabetes mellitus services.

Items	Interviewee type	n (%)
**Facility type**		
Tertiary and specialized hospital	Director or their representative	10 (3.7)
District hospital (DH) [Secondary level]	Civil Surgeon (CS)/Superintendent	58 (21.8)
Upazila Heath Complex (UHC) [Primary level]	Upazila Health and Family Planning Officer (UH&FPO) or Resident Medical Officer (RMO)	124 (46.6)
NGO & Private Hospital	Head of NGO/private hospital	74 (27.8)
***Total***		***266 (100)***
**Facility Ownership**		
Public		192 (72.1)
Private/NGO		74 (27.9)
***Total***		***266 (100)***
**Divisions**		
Dhaka		73 (27.4)
Chittagong		43 (16.2)
Rajshahi		35 (13.2)
Khulna		42 (15.8)
Rangpur		33 (12.4)
Barishal		23 (8.6)
Sylhet		17 (6.4)
***Total***		***266 (100)***

Results are expressed as number and percentages.

### Service availability for diabetes care

Table 3 describes the mean availability of DM services in all four domains stratified by health facilities. Though doctors and specialists in internal medicine were available at almost all the facilities but availability of endocrinologists were inadequate [at tertiary hospitals 60%, only]. Under the domain of basic technologies/equipment, all items were available in most of the facilities, except blood glucose meter. Furthermore, among the diagnostic facilities, urine dipstick- for albumin/ketones tests was comparatively the least available in all tiers of health facilities. Moreover, all essential medicines except Met form in, were unavailable across all facilities, especially injectable insulin (Table 3).

**Table 3:** Mean availability of DM service readiness indicators in different levels of facilities (n = 320).

Domain	Tertiary & specialized hospital (n = 10)	DH (n = 58)	UHC (n = 124)	NGO & PH (n = 74)
**Staff and Guideline**				
Endocrinologist	6 (60)	4 (6.9)	0 (0.0)	8 (10.8)
Medicine doctor	10 (100)	51 (87.9)	45 (36.3)	43 (58.1)
At least one staff member (Nurse, Technician) providing DM services trained in some aspect of DM care	6 (60)	14 (24.1)	41 (33.1)	12 (16.2)
Guidelines for diagnosis & treatment of diabetes mellitus (DM)	8 (80)	39 (67.2)	77 (62.1)	44 (59.5)
**Basic technologies/Equipment**				
Adult weighing scale	10 (100)	57 (98.3)	117 (94.4)	61 (82.4)
Measuring tape/Height Board (used for measuring height)	10 (100)	57 (98.3)	120 (96.8)	68 (92)
Glucometer	10 (100)	57 (98.3)	102 (82.3)	67 (90.5)
Blood pressure measurement device	10 (100)	52 (89.7)	114 (91.9)	67 (90.5)
**Diagnostic facility**				
Blood glucose	10 (100)	57 (98.2)	107 (86.3)	69 (93.2)
Urine dipstick- protein	9 (90)	46 (79.3)	70 (56.9)	63 (85.1)
Urine dipstick- albumin/ ketones	7 (70)	29 (50.0)	32 (25.8)	45 (60.8)
**Essential medicines**				
Glucose 50% injection	5 (50.0)	17 (29.3)	14 (11.3)	21 (28.0)
Sulphonyl urea	5 (50.0)	28 (48.3)	42 (33.9)	32 (43.2)
Metformin 500mg/800mg	7 (70.0)	36 (62.1)	60 (48.4)	42 (56.8)
Injectable Insulin (Insulin regular, Insulin intermediate, Long acting Insulin and Insulin Analogues)	4 (40)	10 (17.6)	6 (4.6)	30 (40.0)

Results are expressed as percentages. TH: Tertiary care hospitals, DH: District hospitals, UHC: Upazila Health Complex, PH: Private Hospital.

### Health facility readiness

**Fig 2:**
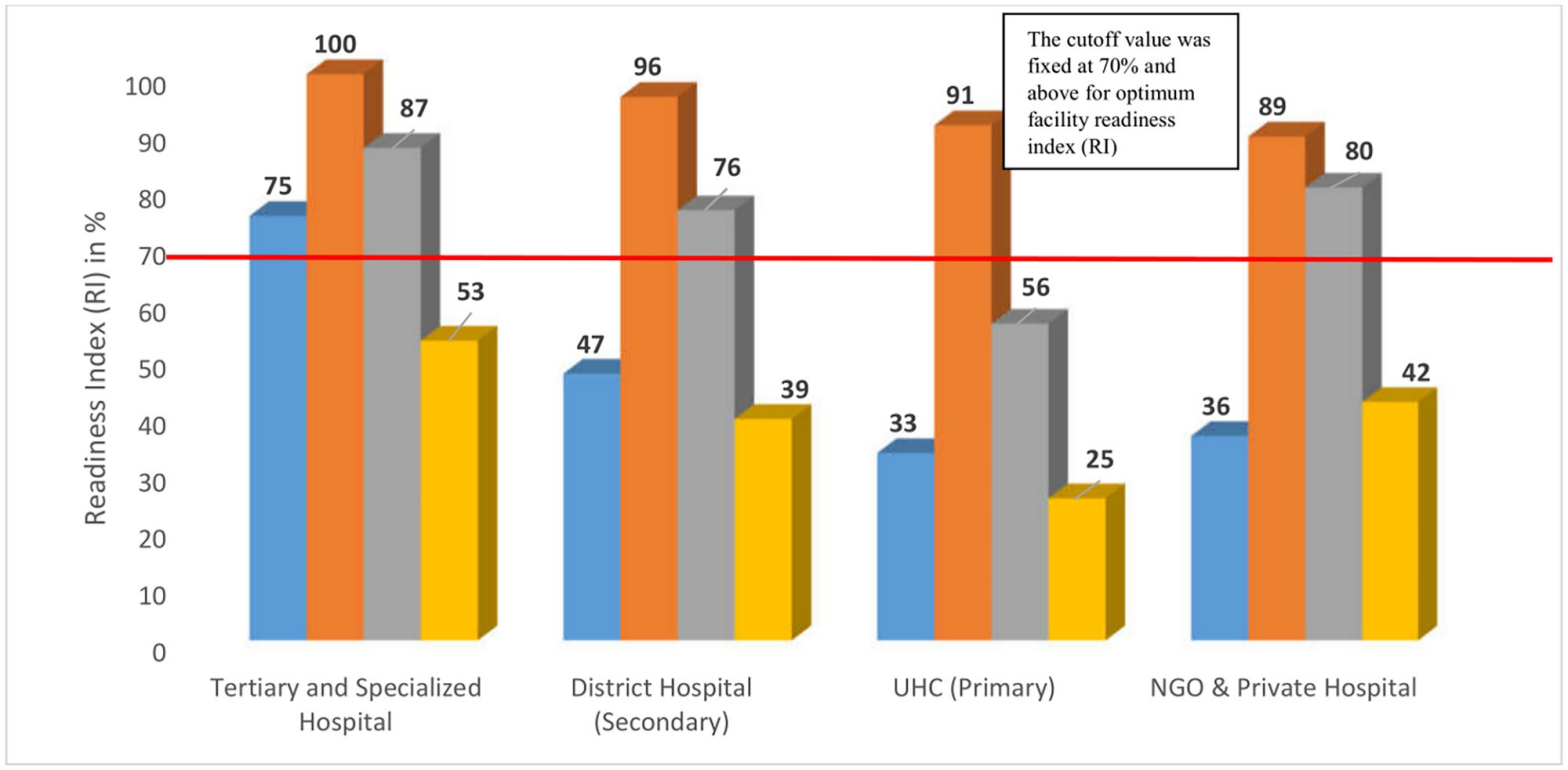
Domain specific DM service readiness index stratified by facility level (the red line indicates the cut off value 70%, above which a hospital is considered to be ’ready’ to provide DM services).

Domain specific DM service readiness index stratified by facility level is shown in Fig 2 where no facility could reach the cutoff point (70%) in all 4 domains. Though tertiary hospitals had crossed the cutoff score of 70% in other 3 domains. However, they lacked in essential medicines (53%). Under the domain of basic equipment, all facilities reached cut off line. RI was considerably low (<50%) in the majority of domain components among the primary and secondary health facilities, particularly in the domains of ‘staffs & guideline’ and ‘essential medicines’. Among all the facilities, readiness index in the staff and guideline domain were low, with the lowest by UHC (33%) (Fig 2).

**Fig 3:**
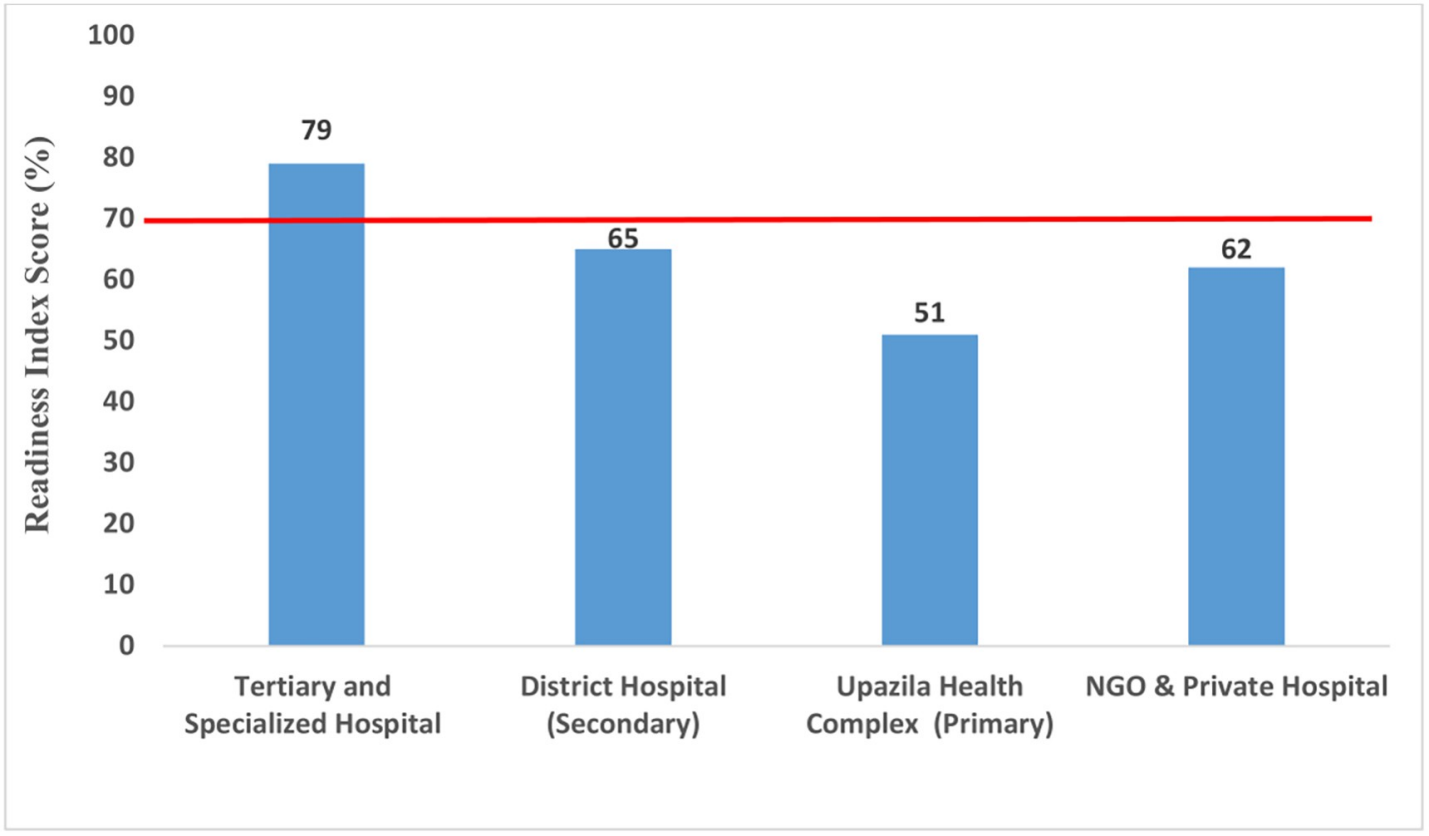
Mean DM service readiness index score for each facility level (the red line indicates the cut off value 70%, above which a facility is considered to be ’ready’ to provide DM services).

Readiness to manage diabetes mellitus was stratified widely at each level of health facility (Fig 3). In line with this, actually no facility (public as well as private) was fully ready to manage DM as none of them could surpass the cut off value in all 4 domains, not even the tertiary level facilities. Though the mean RI was 79% in tertiary level facility, still they lacked in essential medicine. For the rest of the facilities in primary and secondary levels, the mean RI in descending order are- District Hospitals (DH): 65%, Upazila Health Complexes (UHC): 51%. On the other hand, the mean RI of NGO and private hospitals were also below the threshold (62%) (Fig 3).

## Discussion

The present study described a comprehensive scenarioof the service availability and readiness of health facilities to provide diabetes care in the context of Bangladesh and identifiedthe gaps between the facility levels. The major finding of this study is that, among all tiers of health services (primary, secondary, tertiary) in Bangladesh, only tertiary and specialized hospitals were ready to provide DM services. Despite having a high average RI (79%), the availability of essential medicine was inadequate in the tertiary level facilities. Furthermore, the primary level health facilities of Bangladesh (UHC) are not equipped to provide the bare minimum diagnostic facilities for the DM patients. These findings are aligned with numerous studies from LMIC presentation health facilities are not completely ready to provide comprehensive diabetes care services [16,22–25]. Moreover, the study reveals that the NGOs and private facilities have better performance in terms of service availability and readiness in providing diabetic care.

While in comparison with a previous study by T Biswas *et al* in 2016 [16], it was found that the mean RI score of district hospitals and UHCs increased by three-fold (22.2% to 65.0%) and more than two-fold (23.4% to 51.0%), respectively. In terms of readiness for essential medicine, all facilities scored low which is similar to previously conducted study [16]. However, in both studies the readiness index score was found low for training of healthcare providers on diabetes management. It is a well-known fact, trained staff plays a vital role in healthcare services, and thus ensuring specific training for healthcare personnel could assure effective care for NCDs as a whole, and diabetes in particular [26–28]. Moreover, the present study found higher domain specific score for equipment in district hospitals (96%) and UHC (91%) compared with T Biswas’s study (mean domain score: 77.2%). In terms of readiness for essential medicine, all facilities had low accessibility of medicines which are similar to the reference study [16]. In regard to health facility readiness, such as access to drug, as well as training of healthcare workers concerned with diabetes patients, the present study findings are consistent with several other studies conducted in South Asia and Sub-Saharan Africa [29–31].

One more key finding of this study is that the availability of medicine tracer items is significantly poor in all tiers for diabetes management and treatment. This is consistent with the previous studies in Bangladesh that revealed a significant shortage in the availability, and/or an inadequate or limited supply of relevant and essential medicines for diabetes, within the primary healthcare facilities [32]. This indicates, our healthcare system is still not fully prepared to combat diabetes [16], and improvisation is needed in the drug administration policy of the health sector of Bangladesh.

In particular, Bangladesh’s primary and secondary healthcare facilities are found to be under-equipped to provide standard diabetes care with a readiness score lower than the cut-off value of 70%. Traditionally, maternal, child and reproductive health, immunization, and communicable diseases services have been the main focus of primary healthcare system (particularly MCWC, UHCs, FWC/CCs) in Bangladesh [33]. Although, Bangladesh is among the top three countries with regards to number of patients diagnosed with diabetes [34], the provision of diabetic care is under equipped in primary health care level and lacks focus in policy level. Bangladesh government has taken some initiatives regarding NCD care, which includes establishment of a NCD corner in the UHCs [35]. However, in reality, these NCD corners lacked all the domains except basic equipment. Moreover, despite having a national guideline for diabetes management, it is not adequately followed in most of the primary and secondary level healthcare facilities. Effective monitoring and evaluation of services at different level of health facilities, along with capacity development of the healthcare professionals are essential to address these limitations.

Like all other developing countries, Bangladesh is also not prepared for the epidemiological shift that it is experiencing, and therefore, it is not surprising that the country presents low mean readiness score for diabetes care management [36]. Nevertheless, as the burden of diabetes is increasing at an alarming rate, the development of standard diabetic care across all facilities in Bangladesh should come into the priority of the government to achieve universal health coverage [37].

### Strengths and limitations of the study

This study explored a comprehensive scenario of health services for diabetes mellitus management from rural to urban under public and private support public (stratified by all levels) health services for diabetes mellitus management throughout Bangladesh. However, factors such as lack of sampling frame for private healthcare facilities and resource constrain, affected the methodological robustness of the sampling technique. Despite this limitation, the sample size of this study is possibly the largest among other relevant studies, and it was decided upon consultation with experts from Directorate General of Health Services in Bangladesh (DGHS) who validated the sample size for data saturation. The analysis also has limitation as the preparedness at different levels of facilities for private sector couldn’t be reported, nor could a comparative readiness analysis between public and private facilities be performed. Future research could apply a more systematic sampling approach to investigate the differences in services between public and private facilities, as well as variations in services among different levels of private facilities for the management of diabetes. Future studies should look into the underlying causes of service gaps in specific components of the health system, including policy and organizational barriers.

Despite its limitations, the study produced critical evidence on the overall readiness of the country’s health system to manage diabetes and the identification of domains that require strengthening, which can serve as a foundation for developing domain and facility level specific interventions.

## Conclusions

Findings of the present study indicate that in spite of ample investment, Bangladesh is far behind from being ready to manage DM across all tiers of health service provision. Only tertiary hospitals could surpass the cut off value in terms of mean RI score, but they lacked essential medicine when considering domain specific scores. Although the study encourages a positive viewpoint since all tires of health services in Bangladesh have available basic equipment for diabetes management, however the number of trained healthcare personnel and essential medicines were not adequate as for a well-functioning health system. Therefore, to achieve universal health coverage, a well-designed plan to ensure the availability of skilled staffs, adequate medicines, and effective diagnostic facilities at both public and private sectors, particularly in primary and secondary healthcare levels, should be given high priority while developing health policies.

## Supporting information

S1 File Name of participating hospitals or institutes. (PDF)

S2 File Dataset of the cross-sectional national survey to assess service availability and readiness of healthcare facilities to manage diabetes mellitus in Bangladesh. (SAV)
