# Supplementary material for: Assessing service availability and readiness of healthcare facilities to manage diabetes mellitus in Bangladesh: Findings from a nationwide survey
Source: PLoS One. 2022 Feb 16;17(2):e0263259. doi: 10.1371/journal.pone.0263259 (PMC8849622; doi:10.1371/journal.pone.0263259)
Supplement: S1 File — (PDF) [file pone.0263259.s001.pdf]

Sl No	Name of Participating Hospitals/Instritutes
1	National Institute of Cancer Research, Dhaka
2	National Institute of Opthalmology, Dhaka
3	Mymensingh Medical College Hospital, Mymensingh
4	Birdem General Hospital, Dhaka
5	Dhaka Medical College and Hospital, Dhaka
6	Bangobandhu Seikh Mujib Medical University, Dhaka
7	Rajshahi Medical College and Hospital, Rajshahi
8	Rangpur Medical College and Hospital, Rangpur
9	Shahid Tajuddin Ahmad Medical College and Hospital, Gazipur
10	National Institute of Cardiovascular Disease, Dhaka
11	Jhalokathi District Hospital
12	Jamalpur General Hospital
13	Noakhali General Hospital
14	Bhola District Hospital
15	Hobiganj District Hospital
16	Lakshmipur District Hospital
17	Sunamganj District Hospital
18	Patuakhali District Hospital
19	Bandarban District Hospital
20	Narayanganj District Hospital
21	Adunik District Hospital, Netrakona
22	Rajbari General Hospital
23	Adhunik District Hospital, Nilphamary
24	Khulna General Hospital
25	Bagerhat District Hospital
26	Feni District Hospital
27	Sylhet Shahid Shamsuddin Ahmed District Hospital/ (Sylhet District Hospital)
28	Satkhira District Hospital
29	Kishoreganj District Hospital
30	Kushtia District Hospital
31	250 beded Zilla Hospital, Cox's bazar
32	Nawgaon District Hospital
33	Chandpur General Hospital
34	Adhunik Zilla Hospital, Joypurhat
35	Gaibandha District Hospital
36	Lalmonirhat District Hospital
37	Jessore 250 bedded General Hospital
38	Sherpur District Hospital
39	Meherpur 250 bedded Hospital
40	Dinajpur General Hospital
41	District Hospital, Soriatpur(100 beded)
42	Brahmanbaria District Hospital
43	Chuadanga District Hospital

44	District Hospital, Newtown, Madaripur
45	250 beded General Hospital, Gopalganj
46	Adhunik District Hospital, Tetulia
47	Faridpur General Hospital
48	Comilla General Hospital
49	Munshiganj District Hospital
50	General Hospital, Pabna
51	District Hospital, Barguna
52	Districtt Hospital, Kurigram
53	Barishal General Hospital
54	Chittagong General Hospital
55	Khagrachari Adhunik District Hospital
56	Magura District Hospital
57	Muhammad Ali District Hospital, Bogra
58	Moulvibazar District Hospital
59	District Hospital, Jhenaidha
60	250 bed District Hospital, Manikganj
61	100 beded District Hospital, Narsingdi
62	District Hospital, Sirajganj
63	Adhunik District Hospital, Natore
64	Adhunik District Hospital, Chapai Nawabganj
65	Narail District Hospital
66	Panchagarh District Hospital
67	Pirojpur District Hospital
68	Adhunik District Hospital,Gazipur
69	Nagarpur Upazila Health Complex, Tangail
70	Betagi Upazila Health Complex, Barguna
71	Saidpur Upazila Health Complex, Nilphamari
72	Babuganj Upazila Health Complex, Barisal
73	Nolchity Upazila Health Complex, Jhalokathi
74	Aditmari Upazila Health Complex, Lalmonirhat
75	Sundargonj Upazila Health Complex, Gaibandha
76	Sadullapur Upazila Health Complex, Gaibandha
77	Katiyadi Upazila Health Complex, Kishorganj
78	Sharisabari Upazila Health Complex, Jamalpur
79	Gangni Upazila Health Complex, Meherpur
80	Baliadangi Upazila Health Complex,Thakurgaon
81	Nogarkanda Upazila Health Complex, Faridpur
82	Banaripara Upazila Health Complex, Barisal
83	Tajumuddin Upazila Health Complex, Bhola
84	Upazila Health complex mohammadpur, Magura
85	50 bed Upazila Health Complex, Delduar, Tangail
86	Rajnagar Upazila Health Complex, Maulvibazar
87	Upazila Health Complex, Dupchanchia, Bogra

88	Upazila Health Complex, Adamdighi, Bogra
89	Upazila Health Complex, Nachole, Chapainababganj
90	Upazila Health Complex,Chorvodrason, Faridpur
91	Rajapur Upazilla Health Complex, Jhalokathi
92	Assasuni Upozilla Health Complex, Satkhira
93	Fakirhat Upazila Health Complex, Bagerhat
94	Trishal Upazila Health Complex, Mymensigh
95	Phulpur Upazila Health Complex, Mymensigh
96	Melandoho Upazila Health Complex, Jamalpur
97	Mirzagohj Health Complex, Patuakhali
98	Pathorghata Upazila Health Complex, Barguna
99	Kalapara Upazila Health Complex, Patuakhali
100	Upazila Health Complex, Atghoria, Pabna
101	Chakaria Upazila Health Complex, Cox's Bazar
102	Nalitabari Upazila Health Complex, Sherpur
103	Rupganj Upazila Health Complex, Narayanganj
104	Puthiya Upazila Health Complex, Rajshahi
105	Paba Upazila Health Complex, Rajshahi
106	Mirpur Upazila Health Complex, Kushtia
107	Kumarkhali Upazila Health Complex, Kushtia
108	Rajesshori Upazila Health Complex, Kurigram
109	Rajarhat Upazila Health Complex, Kurigram
110	Kalai Upazila Health Complex, Joypurhat
111	Akkelpur Upazila Health Complex, Joypurhat
112	Sapahar Upazila Health Complex, Naogaon
113	Manikchari Upazila Health Complex, Khagrachhari
114	Jagannathpur Upazilla Health Complex, Sunamganj
115	Derai Upazila Health Complex, Sunamganj
116	Rupsha Upazilla Health Complex, Khulna
117	Golapganj Upozilla Health Complex, Sylhet
118	Tongibari Health Complex, Munshiganj
119	Sonargaon Upazila Health Complex, Narayanganj
120	Kamarkhand Upazila Health Complex, Sirajganj
121	Ramu Upazila Health Complex, Cox's bazar
122	Rowangchhari Upazila Health Complex, Bandarban
123	Ruma Upazila Health Complex, Bandarban
124	Dhamrai Upazila Health Complex, Dhamrai
125	Pirgacha Upazilla Health Complex, Rangpur
126	Upazila Health Complex, Lohagara, Narail
127	Upazilla Health Complex, Kauniya, Rangpur
128	Upazila Health Complex, Patnitala, Naogaon
129	Daganbhuyaan Upazila Health Complex, Feni
130	Madhabpur Upazila Health Complex, Hobiganj
131	Kaliganaj Upazila Health Complex, Lalmonirhat

132	Sirajdikhan Upazila Health Complex, Munshiganj
133	Jhikargacha Upazila Health Complex, jhikargacha, Jessore
134	Jaintapur Upazilla Health Complex, Sylhet
135	Damurhuda Upazila Health Complex, Chuadanga
136	Upazila Health Complex, Baniachong, Hobiganj
137	Barhatta Upazila Health Complex, Netrakona
138	Upazila Health Complex, Chirirbandor, Dinajpur
139	Atwari Upazila Health Complex, Panchagarh
140	Akhaura Upazila Health Complex, borobazar, Brahmanbaria
141	Kaharole upazila health Complex, Dinajpur
142	Bajitpur Upazila Health Complex, Kishorganj
143	Nokla Upazila Health Complex, Sherpur
144	Upazila Health Complex, Palash, Narsingdi
145	Shibpur Upazila Health Complex, Narsingdi
146	Jibonnagar Upazila Health Complex, Chuadanga
147	Zanjira Upazila Health Complex, Shariatpur
148	Razoir Upazila Health Complex, Madaripur
149	Kalkini Upazila Health Complex, Madaripur
150	Kotalipara Upazila Health Complex,Gopalganj
151	Tungipara Upazila Health Complex, Gopalganj
152	Mohanganj Upazila Health Complex, Netrakona
153	Pirganj Upazila Health Complex, Thakurgaon
154	Boda Upazila Health Complex, Panchaghar
155	Upazila Health Complex, Goalondo, Rajbari
156	Upazila Health Complex, Pangsa, Rajbari
157	Upazila Health Complex, Borhanuddin, Bhola
158	Upazila Health Complex, Faridganj, Chandpur
159	Domar Upazilla Health Complex, Nilphamari
160	Upazila Health Complex, Savar
161	50 bed Upazila Health Complex, Kaliakair, Gazipur
162	Upazila Health Complex, Kalia, Narail
163	50 bed Upazila Health Complex, Shingair, Manikganj
164	Kaligong Upazila Health Complex, Jhenaidah
165	Upazila Health Complex, Kaliganj, Gazipur
166	Hathazari Upazila Health Complex, Chittagong
167	Anowara Upazila Health Complex, Chittagong
168	Upazila Health Complex,sreepur, Magura
169	Shreemangal Upazila Health Complex, Maulvibazar
170	50 bed Upazila Health Complex, Ghior, Manikganj
171	Phultola Upazila Health Complex, Khulna
172	Mujibnagar Upazila Health Complex,Meherpur
173	Vandaria Upazila Health Complex, Pirojpur
174	Bagatipara Upazila Health Complex, Natore
175	Upazila Health Complex,Raiganj, Sirajganj

176	Upazila Health Complex,Ishwardi, Pabna
177	Gurudashpur Upazila Health Complex, Natore
178	Companiganj Upazila Health Complex, Noakhali
179	Begumganj Upazila Health Complex, Noakhali
180	Haimchar Upazila Health Complex, Chandpur
181	Choddagram Upazila Health Complex, Comilla
182	Matiranga Upazila Health Complex, Khagrachari
183	Upazila Health Complex kotchadpur, Jhenaidah
184	Monirampur Upazila Health Complex, Jossore
185	Chagalnaya Upazila Health Complex, Feni
186	Gomastapur Upazila Health Complex, Chapai nawabganj
187	Burichang Upazila Health Complex, Comilla
188	Tala Upazila Health Complex, Paikgacha road, Satkhira
189	Mongla Upazila Health Complex, Bagerhat
190	Mathbaria Upazila Health Complex, Pirojpur
191	Ashuganj Upazila Health Complex, Brahmanbaria
192	Upazila Health Complex(50 beded), Nariya, Shariatpur
193	Impact Jibon Mela Hospital, Meherpur
194	Islami Bank Hospital, Rajshahi
195	Islami Hospital, Chapai Nawabganj
196	Green Life General Hospital, Chapai Nawabganj
197	Lakesite Hospital pvt., Rangamati
198	Seba Diagnostic and Nursing Home, Dinajpur
199	New Square Hospital, Thakurgaon
200	Kishoreganj Clinic, Kishorganj
201	Chenghi kashbon Hospital & maternity clinic, Khagrachari
202	Sono Hospital, Courtpara, Kushtia
203	Islami Hospital & diagnostic center, hospital road, Chuadanga
204	Feni Diabetic Hospital
205	Modern general Hospital and Diagnostic Centre, Joypurhat
206	Medilab health centre limited, Kishorganj
207	Sonargaon sheba general Hospital,Narayanganj
208	Mirpur General Hospital and Diagnostic Centre, Dhaka
209	Brighton Hospital, Sylhet
210	City Hospital, Pathukhali
211	Upasham General Hospital, Feni
212	Liza clinic and digonostic center, Rajbari
213	Janata Hospital & Diagnostic Center, Jessore
214	Gaibandha Clinic
215	United General Hospital, Jamalpur
216	Rahat Anowar Hospital, Chandmari, Barishal
217	Central Clinic and Diagnostic Centre, Kurigram
218	Rahat Anowar Hospital
219	Ideal Clinic & Diagnostic Complex, Fakirhat, Bagerhat

220	Janoseba Clinic and Diagnostic Center, Satkhira
221	Khulna Health Garden, Khulna
222	Holy Diagnostic Centre, Naogaon
223	Emanuel Medical Center, Bandarban
224	Hill view Hospital & Diagnostic Center, Bandarban
225	Thakurgaon Diabetic and Shasthoseba Hospital
226	Nipun Clinic and Diagnostic, Soriatpur
227	Seba Clinic and Diagnostic Center, Munshiganj
228	Sherpur doctors Hospital
229	Farabi General Hospital, Dhaka
230	Babu Chowdhury General Hospital and Diagnostic Centre, Madaripur
231	Dr Forid Memorial Hospital, Gopalganj
232	Group Clinic and Diagnostic Centre, Gopalganj
233	New Life Care Hospital limited, Habiganj
234	Central Hospital Private Limited, Chandpur
235	Central Diagonotic Centre and Hospital,Brahmanbaria
236	Rangpur Adhunik Hospital
237	Avicenna Hospital, Sirajganj
238	Nilphamari diabetic samiti Hospital
239	Rongdhonu General Hospital, Mymensingh
240	Sono nursing home, Meherpur
241	Central Hospital, Narsingdi
242	Chittagong Metropolitan Hospital
243	Shimla Hospital & Diagnostic Center, Pabna
244	Lakshmipur Adhunik Hospital
245	Central Hospital, Noakhali
246	Kalia Surgical Clinic, Narail
247	Greenview diagnostic complex, Magura
248	Sonia Nursing Home, Tangail
249	Seba Clinic, Chapai Nawabganj
250	Modern Clinic, Chapai Nawabganj
251	Hazrat shahjalal (RA) Hospital and Pathology Center. Manikganj
252	Fatema Memorial Hospital, Bhola
253	Al Raji Clinic and Diagnostic Center, Dhaka
254	Jonosheba Hospital, Natore
255	Shamsunnahar Clinic, Bogra
256	Islamia Digital Diagnostic and General Hospital, Naogaon
257	Jhenaidha Prince Hospital Ltd
258	Albaraka Hospital and Diagnostic Centre, Brahmanbaria
259	Moulvibazar Poly Clinic
260	Medi Hospital, Comilla
261	Sodesh Hospital pvt. LTD, Mymensingh
262	Muslim Aid Hospital, Pirojpur
263	Rowshon Clinic and General Hospital, Panchagarh

264	City General Hospital, Panchagarh
265	Karomtala Christian Hospital, Gazipur
266	Brac Health Center, Sunamganj
